# Hyperglycaemia, adverse outcomes and impact of intravenous insulin therapy in patients presenting with acute ST‐elevation myocardial infarction in a socioeconomically disadvantaged urban setting: The Montefiore STEMI Registry

**DOI:** 10.1002/edm2.89

**Published:** 2019-08-14

**Authors:** Sanyog G. Shitole, Vankeepuram Srinivas, Julia L. Berkowitz, Tina Shah, Michael J. Park, Samuel Herzig, Anne Christian, Neeral Patel, Xiaonan Xue, James Scheuer, Jorge R. Kizer

**Affiliations:** ^1^ San Francisco Veterans Affairs Health Care System and University of California San Francisco San Francisco CA USA; ^2^ Montefiore Health System Bronx NY USA; ^3^ Geisel School of Medicine at Dartmouth Hanover NH USA; ^4^ Albert Einstein College of Medicine Bronx NY USA

**Keywords:** hyperglycaemia, outcomes, STEMI

## Abstract

**Background:**

Hyperglycaemia occurs frequently in ST‐elevation myocardial infarction (STEMI) and is associated with poor outcomes, for which continuous insulin infusion therapy (CIIT) may be beneficial. Information is limited regarding hyperglycaemia in acute STEMI affecting urban minority populations, or how CIIT fares in such real‐world settings.

**Methods and results:**

We assembled an acute STEMI registry at an inner‐city health system, focusing on patients with initial blood glucose ≥180 mg/dL to determine the impact of CIIT vs usual care. Clinical and outcomes data were added through linkage to electronic records. Inverse‐probability‐of‐treatment weighting using propensity scores (PS) was used to compare CIIT vs no CIIT. The 1067 patients included were mostly Hispanic or African American; 356 had blood glucose ≥180 mg/dL. Such pronounced hyperglycaemia was related to female sex, minority race‐ethnicity and lower socioeconomic score, and associated with increased death and death or CVD readmission. CIIT was preferentially used in patients with marked hyperglycaemia and was associated with in‐hospital hypoglycaemia (21% vs 11%, *P* = .019) and, after PS weighting, with increased in‐hospital (RR 3.23, 95% CI 0.94, 11.06) and 1‐year (RR 2.26, 95% CI 1.02, 4.98) mortality. No significant differences were observed for death at 30 days or throughout follow‐up, or death and readmission at any time point.

**Conclusions:**

Pronounced hyperglycaemia was common and associated with adverse prognosis in this urban population. CIIT met with selective use and was associated with hypoglycaemia, together with increased mortality at specific time points. Given the burden of metabolic disease, particularly among race‐ethnic minorities, assessing the benefits of CIIT is a prerogative that requires evaluation in large‐scale randomized trials.

## Graphical abstract

Weighted risk estimates (with 95% confidence intervals) for the main outcomes at different time points for CIIT versus no CIIT (no CIIT is the referent) additionally adjusting for smoking status. All risk estimates are risk ratios except for those corresponding to comparisons through follow‐up (labelled overall), which are hazard ratios. CIIT=Continuous insulin infusion therapy, CVD=Cardiovascular disease.

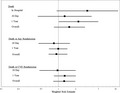

## 1 BACKGROUND

There is strong and consistent evidence that patients with ST‐segment elevation myocardial infarction (STEMI) who present with elevated blood sugar have increased short‐ and long‐term mortality rates.^1^, ^2^, ^3^, ^4^ Hyperglycaemia has also been linked in this setting to no reflow after percutaneous coronary intervention (PCI), to increased infarct size, as measured by serum myocardial markers and magnetic resonance imaging, and to a greater incidence of cardiogenic shock and heart failure.^5^, ^6^, ^7^ Hyperglycaemia appears to be a marker of poor outcomes, irrespective of presence or absence of a prior history of diabetes mellitus and may even be more consequential in those STEMI patients who do not have a history of hyperglycaemia.^8^, ^9^

A variety of glucose measurements following presentation with STEMI, whether during the first 24 hours after admission or during hospitalization after the acute event, have been cited as having prognostic significance.^10^, ^11^ Haemoglobin A1c (HbA1c) during hospital admission has also been noted to be predictive of long‐term events in STEMI.^12^ It had been assumed that lowering of blood sugar would afford protection in hyperglycaemic STEMI patients, and the first Diabetes Mellitus Insulin‐Glucose Infusion in Acute Myocardial Infarction (DIGAMI) Trial seemed to confirm that concept.^13^ Evidence suggests that judicious treatment of hyperglycaemia in acute myocardial infarction (AMI) patients improves outcomes.^14^ Numerous protocols have been employed, along with a variety of glycemic targets, but these interventions have not yielded consistent results. The original DIGAMI trial differs from DIGAMI 2 in that patients entered the first study with higher glucose and HbA1c values and had greater declines in blood glucose over the first 24 hours than in the second study.^15^ In general, there has been no consistency among and within studies in achieving a safe, mildly hyperglycaemic level. In considering this, the American College of Physicians guidelines in 2011 concluded that quality of the studies related to this issue is not adequate to make a recommendation for therapy.^16^ More recent recommendations from experts and professional societies consider the risks of hypoglycaemia noted with tight glucose control in surgical or critically ill patients to call for lowering blood glucose levels to safe but reasonable levels in the AMI setting.^17^, ^18^, ^19^ Generally, the protocol has been to use intravenous insulin, with frequent monitoring to bring blood glucose levels into the 140‐180 mg/dL range.^17^ The 2013 American College of Cardiology Foundation/American Heart Association STEMI Guidelines concur with lowering blood glucose to <180 mg/dL while avoiding hypoglycaemia.^18^

In this context, scant data reflecting real‐world experience on the application of continuous insulin infusion therapy (CIIT) in patients with STEMI are available, particularly among race‐ethnic minorities from socioeconomically disadvantaged urban centres in the United States. Moreover, despite susceptibility to metabolic dysregulation among such race‐ethnic minority populations, there is little information on the extent, correlates and consequences of STEMI‐related hyperglycaemia among such vulnerable sociodemographic groups.^20^ Here, we report on a large urban health system's experience with implementation of a CIIT protocol geared to control hyperglycaemia in patients with acute STEMI, leveraging concurrent creation of a STEMI registry and subsequent linkage to administrative and clinical databases to characterize the distribution and severity of hyperglycaemia, acute treatment patterns, and associated short‐ and long‐term outcomes in this high‐risk population.

## 2 METHODS

### 2.1 Study procedures and population

The Montefiore STEMI Registry included all STEMI patients coming for treatment from May 2008 to December 2014 to Montefiore Health System (MHS) and providing informed consent. The original primary goal of the registry was to evaluate the impact of a CIIT protocol used for patients presenting with an initial glucose ≥150 mg/dL. The target glucose levels with therapy were 80‐120 mg/dL. In 2010, the initial glucose value was changed to ≥180 mg/dL with a glucose target of 100‐180 mg/dL. The CIIT protocol employed an infusion pump administering 100 units of regular insulin in 100 mL normal saline with glucose evaluated by hourly monitoring using finger stick (FS). The insulin infusion rate was varied according to the FS glucose results. In the event that hypoglycaemia was detected, a strict treatment protocol was initiated. After 24 hours, the insulin infusion could be stopped and switched to subcutaneous insulin if glucose levels were at goal. The use of this protocol was encouraged, but optional. Alternatively, ‘usual care’ could include ad hoc intravenous or subcutaneous insulin as guided by periodic FS or blood glucose levels and a recommended sliding scale. Patients who presented in cardiogenic shock, defined by clinical and hemodynamic criteria,^21^ or had end‐stage renal disease on admission, defined as need for acute or chronic dialysis, were not eligible for the CIIT protocol and are excluded from the current analysis. In introducing this protocol, educational information was provided to cardiology fellows, medical house staff and nurses in the Coronary Care Units (CCU) of our two major sites, the Moses and Weiler divisions of MHS. The initial FS or venous blood glucose was obtained in the Emergency Room or Cardiac Catheterization Laboratory (CCL), and the first value was used to decide on therapy. The CIIT protocol, when used, could be initiated in the CCL or CCU. Initially, special intake forms were used to record patient information, which was gathered from direct interviews with patients and from chart reviews by nurse abstractors. Information included sociodemographic characteristics, medical history, physical examination, laboratory results and other diagnostic test findings. In‐hospital characteristics such as blood and FS glucose values, and blood values for cardiac markers were obtained from a proprietary database system, Looking Glass Clinical Analytics (LGCA; Streamline Health). LGCA is an interactive software application in use across MHS, which integrates clinical and administrative data to allow evaluation of healthcare quality.^22^ Information collected by nurse abstractors on clinical, laboratory and imaging data was supplemented using LGCA. Where missing data on clinical characteristics were not directly queriable by LGCA, trained physician abstractors undertook direct review of electronic medical records (EMR). Cardiac catheterization data were obtained from an electronic database containing standardized angiographic and procedural information reported to New York State. Mortality data were obtained by linkage to the National Death Index (NDI). LGCA was also used to capture rehospitalizations at MHS and the adjacent Jacobi Medical Center and North Central Bronx Hospital (together forming the North Bronx Health Network [NBHN]) after the index STEMI hospitalization. The STEMI registry protocol was approved by the Institutional Review Board of the Albert Einstein College of Medicine.

### 2.2 Definition of covariates

Race‐ethnicity was defined by self‐report. Summary socioeconomic score was calculated using a published algorithm.^23^ Body mass index (BMI) was derived as weight (kg) divided by the square of height (m^2^). Hypertension, diabetes and dyslipidaemia were based on self‐reported or documented history, or treatment with corresponding medications. Current smoking was defined as any cigarette use in the past 30 days. Heavy alcohol use was defined by a history of alcohol abuse or consumption of more than 14 drinks/wk in men and 7 drinks/wk in women. Cocaine use was defined as self‐reported use in the past 4 weeks or a positive urine cocaine test.^24^ Human immunodeficiency virus (HIV) infection was defined by a positive HIV ELISA or a positive HIV viral load and confirmed through linkage to the MHS Center for AIDS Research database. Prior cardiovascular disease (CVD) included coronary heart disease, stroke or peripheral arterial disease. Both prior CVD and prior heart failure (HF) were assessed from patient history or clinical information in the medical record. The TIMI risk score was calculated based on a published algorithm.^25^ Hypoglycaemia was defined as a blood glucose ≤70 mg/dL.^26^ Stress hyperglycaemia was defined as an in‐hospital HbA1c <6.5%, a negative history of diabetes, and an initial glucose value ≥180 mg/dL. Critical coronary disease was defined as ≥70% luminal narrowing of the left anterior descending (LAD), left circumflex or right coronary artery or its branches or ≥50% narrowing of the left main coronary artery. Left ventricular ejection fraction (LVEF) was obtained from the left ventriculogram at cardiac catheterization or, in its absence, from the earliest echocardiogram.

### 2.3 End‐point ascertainment and definitions

Readmission data to MHS were obtained from LGCA. Because a substantial number of STEMI admissions would be direct transfers from the Emergency Rooms of the adjacent NBHN and therefore might subsequently be hospitalized there, readmissions of STEMI patients initially transferred from NBHN were also analysed using NBHN’s EMR. Mortality statistics through 2015 were obtained from the NDI. The primary outcome variables were death, death or any rehospitalization, and death or CVD rehospitalization. Apart from in‐hospital death, 30‐day, 1‐year and long‐term follow‐up outcomes for death or its composites are reported. CVD rehospitalization comprised myocardial infarction, revascularization, stroke, HF, ventricular tachycardia/ventricular fibrillation and atrial fibrillation/flutter, based on appropriate Current Procedural Terminology, Fourth Edition (CPT4), codes or discharge International Classification of Diseases, Ninth Revision (ICD9), codes in the primary position, as previously reported.^24^

### 2.4 Statistical analysis

For analysis and presentation, patients were divided into groups based on the initial blood glucose value as: Group 1, initial glucose <140 mg/dL; Group 2, 140‐179 mg/dL (mild elevation); and Group 3, ≥180 mg/dL (pronounced elevation). Group 3 was further subdivided into Group 3A, patients treated with CIIT, and Group 3B, patients not treated with CIIT. This report focuses on the characteristics and outcomes of glycemic categories, and on the comparison of the subgroups defined by CIIT treatment. Continuous variables are described as median and interquartile range, while categorical variables are presented as count and per cent. Comparisons of continuous variables applied the Wilcoxon rank‐sum test, while those of categorical variables and outcomes used the chi‐square or Fisher's exact test, as appropriate. To compare adjusted risks of events at 30 days and 1 year, we performed relative risk regression using a Poisson working model with a log‐link function and robust standard errors. In the case of in‐hospital deaths, the number of events was too low to permit multivariable adjustment. Comparison of times to events over the entire period of follow‐up was performed with Cox proportional hazards models. For the comparison of glycemic categories, adjustment was undertaken by covariates selected based on known or apparent associations with post‐STEMI outcomes. An initial model adjusted for age, sex and race‐ethnicity. A subsequent model adjusted additionally for site, socioeconomic score, BMI, smoking, heavy alcohol use and HIV status. The proportional hazards assumption was tested by Schoenfeld residuals, which revealed no violations.

For the analysis of CIIT groups (3A and 3B), standardized differences were used to compare the balance in measured baseline covariates between those who received the treatment and those who did not, an approach known to be less sensitive to sample size compared to the conventional *P*‐value approach.^27^ As sizable standardized differences (>20%) were observed, a multi‐step approach was taken to address potential confounding by such differences. Using logistic regression, we first created a propensity score (PS) for receiving CIIT among the cohort of patients with initial glucose ≥180 mg/dL using the following covariates: age, sex, race‐ethnicity, summary socioeconomic score, hypertension, diabetes, dyslipidaemia, cocaine use, smoking, prior CVD, prior HF, HIV status, home medications (calcium channel blockers, renin‐angiotensin‐aldosterone system [RAAS] antagonists, statins, thienopyridines, antihyperglycaemic agents), arrhythmia, site, whether the patient was transferred for care, initial glucose and white blood cell (WBC) count. We then applied inverse‐probability‐of‐treatment weighting (IPTW) to the study cohort using the PS.^28^ Next, we assessed how well the PS balanced the treated and untreated groups by calculating the weighted standardized difference in baseline characteristics between these groups. As the high standardized differences observed previously were largely reduced with adjustment by PS weight, we used IPTW to control for potential confounding. We adopted the IPTW approach in the relative risk regression models for the short‐term (30 day and 1 year) outcomes and Cox models for time to event for longer‐term outcomes.^29^ Since smoking was the only variable for which the weighted standardized difference increased to a substantial level (18%) from its crude value, it was additionally included in the regression model. Analyses were performed with SAS, version 9.4 (SAS Institute) and STATA, version 15 (StataCorp LLC). Two‐tailed *P* < .05 defined statistical significance.

## 3 RESULTS

### 3.1 General characteristics

Table 1 presents the sociodemographic and clinical characteristics of the study population, both overall, and stratified by admission glucose values. In the entire sample, two‐thirds of patients were male, and a majority was Hispanic or non‐Hispanic black. There were high frequencies of hypertension, diabetes, dyslipidaemia and current smoking. Median door‐to‐balloon time for nontransfers was slightly over 1 hour, with ninety per cent of those catheterized within 24 hours undergoing percutaneous revascularization. As compared to Group 1, patients in Group 3 were older, less commonly male or non‐Hispanic white and had lower socioeconomic score. They also had higher prevalence of cardiovascular risk factors, and more frequently were taking aspirin. Group 3 patients had greater BMI, but smoked less and reported less alcohol use. They were less frequently HIV seropositive than patients in Group 1. In keeping with their risk profile, Group 3 patients used more antihyperglycaemic agents and RAAS antagonists. On admission, Group 3 patients had higher TIMI risk scores than patients in the lower glycemic categories. In addition, Group 3 patients showed higher median admission heart rate and initial troponin level, with lower LVEF, higher frequency of the LAD artery as the culprit vessel and longer length of stay in comparison with Groups 1 and 2.

**Table 1:** Baseline characteristics of the cohort

Characteristic*	Entire cohort (n = 1067)	Group 1 (n = 432)	Group 2 (n = 281)	Group 3 (n = 354)
Age, y	59 (50, 69)	58 (50, 67)	59 (50, 70)	59 (51, 69)^#^
Males, n (%)	721 (67.6)	311 (71.9)	190 (67.6)	220 (62.2)^#^
Race‐ethnicity, n (%)				
Non‐hispanic white	237 (22.2)	112 (25.9)	65 (23.1)	60 (16.9)^#^
Hispanic	402 (37.7)	144 (33.3)	105 (37.4)	153 (43.2)^#^
Non‐hispanic black	215 (20.2)	83 (19.2)	58 (20.6)	74 (20.9)^#^
Other	213 (19.9)	93 (21.5)	53 (18.9)	67 (18.9)^#^
Summary socioeconomic score	−2.2 (−5.3, −0.8)	−2.0 (−4.9, −0.6)	−2.0 (−5.1, −0.6)	−2.7 (−5.8, −1.1)^#^, **
Hypertension, n (%)	702 (65.8)	257 (59.4)	182 (64.8)	263 (74.3)^#^, **
Diabetes, n (%)	348 (32.6)	43 (9.9)	66 (23.5)^#^	239 (67.5) ^#^, **
Dyslipidaemia, n (%)	571 (53.5)	209 (48.4)	142 (50.5)	220 (62.2) ^#^, **
Cocaine use, n (%)	61 (5.7)	25 (5.8)	21 (7.5)	15 (4.2)
Current smoker, n (%)	404 (37.9)	195 (45.1)	102 (36.6)^#^	107 (30.2)^#^
Heavy alcohol use, n (%)	106 (9.9)	55 (12.7)	25 (8.9)	26 (7.3)^#^
Family history of CAD, n (%)	327 (30.9)	132 (30.8)	88 (31.4)	107 (30.7)
Prior CVD, n (%)	261 (24.5)	99 (22.9)	74 (26.3)	88 (24.9)
Prior HF, n (%)	45 (4.2)	16 (3.7)	13 (4.6)	16 (4.5)
HIV infected, n (%)	29 (2.7)	17 (3.9)	7 (2.5)	5 (1.4)^#^
Home medications, n (%)				
Aspirin	337 (31.6)	120 (27.8)	89 (31.7)	128 (36.2)^#^
Beta‐blocker	300 (28.1)	111 (25.7)	79 (28.1)	110 (31.1)
RAAS antagonist	317 (29.7)	103 (23.8)	79 (28.1)	135 (38.1) ^#^, **
Statin	339 (31.8)	126 (29.1)	87 (30.9)	126 (35.6)
Thienopyridine	99 (9.3)	36 (8.3)	26 (9.3)	37 (10.5)
Diabetes home medications, n (%)				
Oral hypoglycaemics	207 (19.4)	26 (6.0)	40 (14.2)^#^	141 (39.8) ^#^, **
Insulin	123 (11.5)	20 (4.6)	15 (5.3)	88 (24.9) ^#^, **
Body mass index, kg/m^2^	28.2 (25.3, 31.6)	27.9 (25.2, 31.2)	27.8 (25.0, 31.5)	28.9 (25.7, 32.3) ^#^, **
Heart rate, beats per minute	79 (68, 90)	77 (67, 88)	78 (68, 89)	82 (71, 94) ^#^, **
Killip class, n (%)				
I	977 (91.6)	406 (93.9)	255 (90.8)	316 (89.3)
II	60 (5.6)	17 (3.9)	18 (6.4)	25 (7.1)
III	30 (2.8)	9 (2.08)	8 (2.9)	13 (3.7)
LBBB, n (%)	16 (1.5)	5 (1.2)	6 (2.1)	5 (1.4)
Non‐sinus rhythm, n (%)	65 (6.1)	28 (6.5)	16 (5.7)	21 (5.9)
Presenting hospital, n (%)				
Moses	538 (50.4)	224 (51.9)	139 (49.5)	175 (49.4)
Weiler	529 (49.6)	208 (48.2)	142 (50.5)	179 (50.6)
Transfer from another facility, n (%)	250 (23.4)	110 (25.5)	56 (19.9)	84 (23.7)
TIMI STEMI risk score	3 (2, 5)	2 (1, 4)	3 (2, 5)^#^	3 (2, 5) ^#^, **
Initial glucose, mg/dL	151 (122, 210)	117 (105, 128)	157 (148, 166)^#^	246 (210, 311) ^#^, **
HbA1c, %	6.0 (5.7, 7.8)	5.7 (5.5, 6.0)	6.0 (5.7, 6.5)^#^	8.2 (6.7, 10.3) ^#^, **
Peak creatine kinase, u/L	1450 (683, 2865)	1331 (620, 2474)	1637 (696, 3225)^#^	1476 (722, 3083)^#^
Peak troponin T, ng/mL	4.2 (1.9, 8.1)	3.8 (1.7, 7.3)	4.1 (1.8, 8.4) ^#^	4.8 (2.2, 9.3)^#^
Initial serum creatinine, mg/dL	0.9 (0.8, 1.2)	0.9 (0.8, 1.1)	1.0 (0.8, 1.2)	0.9 (0.8, 1.2)
Door‐to‐balloon time^†^, minutes	64 (45, 83)	65 (43, 86)	64 (48, 83)	63 (45, 80)
No. critically diseased vessels^‡^, n (%)				
0	83 (8.1)	38 (9.1)	14 (5.2)	31 (9.1)
1	489 (47.7)	204 (49.0)	144 (53.1)	141 (41.6)
2	290 (28.3)	114 (27.4)	68 (25.1)	108 (31.9)
3	164 (15.9)	60 (14.4)	45 (16.6)	59 (17.4)
LVEF, (%)	50 (40, 60)	50 (40, 60)	50 (40, 60)	48 (38, 59)^#^
Culprit vessel^‡^				
Left anterior descending	472 (46.0)	179 (43.0)	119 (43.9)	174 (51.3)^#^
Left circumflex	110 (10.7)	47 (11.3)	32 (11.8)	31 (9.1)^#^
Right coronary artery	439 (42.8)	190 (45.7)	119 (43.9)	130 (38.4)^#^
Percutaneous coronary intervention^‡^	924 (90.1)	375 (90.1)	248 (91.5)	301 (88.8)
CABG during hospitalization, n (%)	45 (4.2)	15 (3.5)	14 (4.9)	16 (4.5)
Length of stay, days	4 (3, 6)	4 (3, 5)	4 (3, 6)	4 (3, 7) ^#^, **
Newly diagnosed diabetes^§^, n (%)	43 (5.9)	5 (1.3)	9 (4.2)^#^	29 (8.2) ^#^, **
Medications at discharge^||^, n (%)				
Aspirin	1027 (98.9)	419 (98.6)	275 (100.0)	333 (98.2)**
Beta‐blocker	974 (93.7)	398 (93.7)	256 (93.1)	320 (94.4)
RAAS antagonist	780 (75.1)	322 (75.8)	208 (75.6)	250 (73.8)
Statin	999 (96.2)	405 (95.3)	268 (97.5)	326 (96.2)
Thienopyridine	979 (94.2)	406 (95.5)	259 (94.2)	314 (92.6)
Diabetes medications^||^, n (%)				
Oral hypoglycaemics	181 (17.4)	23 (5.4)	39 (14.2)^#^	119 (35.1)^#^, **
Insulin	169 (16.3)	20 (4.7)	15 (5.5)	134 (39.5)^#^, **

Abbreviation: CABG, coronary artery bypass graft surgery; CAD, coronary artery disease; HF, heart failure; CVD, cardiovascular disease; LBBB, left bundle branch block; LVEF, left ventricular ejection fraction; PCI, percutaneous coronary intervention; RAAS, renin‐angiotensin‐aldosterone system; STEMI, ST‐segment elevation myocardial infarction; TIMI, thrombolysis in myocardial infarction; Group 1 = initial glucose <140 mg/dL; Group 2 = initial glucose 140‐179 mg/dL; Group 3 = initial glucose ≥180 mg/dL.

* Median and interquartile range for continuous variables.

^†^ Only for nontransfers undergoing PCI.

^‡^ Only for those with catheterization performed within 24 h.

^§^ Only among nondiabetics at presentation.

^||^ Only for those surviving to discharge.

^#^ *P* < .05 when compared with Group 1.

** *P* < .05 when compared with Group 2.

### 3.2 Overall outcomes by glycemic category

Overall, the study cohort had a median follow‐up of 4.6 years, with a maximum of 7.6 years. For deaths in‐hospital and at 30 days, there were too few events for multivariable analysis between glycemic groups, but unadjusted comparisons showed deaths to be significantly more frequent in Group 3 than Group 1 (in‐hospital, 15 [4%] vs 7 [2%], *P* = .027; 30 days, 18 [5%] vs 9 [2%], *P* = .022). The corresponding proportions of deaths in Group 2 at either time point (6 [2%] in‐hospital and 6 [2%] at 30 days) did not differ significantly from the other groups.

Table 2 shows event rates for death at later time points, and for death and readmission starting at 30 days, along with corresponding risk estimates for the higher glycemic groups after adjustment for demographic factors alone (Model 1), or demographic and behavioural/clinical risk factors (Model 2). Significant associations were observed for Group 3, but not Group 2, in comparison with Group 1 with respect to certain outcomes at specific time points. For all‐cause mortality, Group 3 had over a 2‐fold higher risk at 1 year as compared with Group 1 after adjustment for Model 2 covariates, but there was no significant association during overall follow‐up. For death or CVD readmission, Group 3 had a roughly 60% higher adjusted risk (Model 2) in comparison with Group 1 that was significant, or nearly so, across all three time points. By contrast, no significant relationship was seen among glycemic groups for the outcome of death or readmission for any cause.

**Table 2:** Adjusted models for different events in the three groups

Event	Model	Group 2 vs Group 1		Group 3 vs Group 1	
		Risk estimate* (95% CI)	*P* value	Risk estimate* (95% CI)	*P* value
Death					
At 1 year	1	0.97 (0.53, 1.77)	.925	1.63 (0.98, 2.72)	.059
	2	1.25 (0.67, 2.32)	.482	2.27 (1.33, 3.88)	.003
Entire follow‐up	1	0.84 (0.55, 1.26)	.391	1.26 (0.88, 1.81)	.209
	2	0.90 (0.59, 1.38)	.643	1.44 (0.98, 2.13)	.066
Death or any readmission					
At 30 days	1	0.98 (0.69, 1.39)	.902	0.97 (0.69, 1.36)	.872
	2	0.99 (0.69, 1.43)	.998	1.01 (0.70, 1.44)	.964
At 1 year	1	1.16 (0.97, 1.39)	.103	1.10 (0.93, 1.31)	.261
	2	1.16 (0.97, 1.40)	.110	1.14 (0.96, 1.36)	.144
Entire follow‐up	1	1.12 (0.92, 1.37)	.269	1.16 (0.96, 1.40)	.114
	2	1.13 (0.92, 1.38)	.245	1.18 (0.97, 1.44)	.093
Death or CVD readmission					
At 30 days	1	0.99 (0.57, 1.72)	.960	1.66 (1.04, 2.65)	.033
	2	0.97 (0.55, 1.71)	.926	1.65 (0.99, 2.74)	.052
At 1 year	1	1.03 (0.75, 1.41)	.844	1.60 (1.24, 2.06)	<.001
	2	1.10 (0.81, 1.51)	.539	1.68 (1.29, 2.18)	<.001
Entire follow‐up	1	1.08 (0.83, 1.41)	.560	1.59 (1.26, 2.01)	<.001
	2	1.11 (0.85, 1.45)	.462	1.64 (1.28, 2.09)	<.001

Abbreviations: BMI, body mass index; CI, confidence interval; CVD, cardiovascular disease; HIV, human immunodeficiency virus; SSS, summary socioeconomic score; Group 1 = initial glucose <140 mg/dL; Group 2 = initial glucose 140‐179 mg/dL; Group 3 = initial glucose ≥180 mg/dL.

* All risk estimates are risk ratios except for those corresponding to comparisons through follow‐up, which are hazard ratios; Model 1 adjusts for age, sex, race‐ethnicity; Model 2 adjusts for Model 1, site (Moses vs Weiler), SSS, BMI, current smoking, heavy alcohol use, HIV status.

### 3.3 Characteristics of Groups 3A and 3B

Table 3 shows the results for hyperglycaemic patients treated with CIIT vs those in whom the protocol was not employed (Group 3A vs 3B). Group 3A had a higher initial glucose, poorer median socioeconomic score, more frequent history of and treatment for diabetes and was more likely to be seen at the Moses site. Crude standardized differences frequently exceeded 20%, with the largest observed values seen for prevalent diabetes, hospital site and initial glucose level. After PS‐ adjustment, weighted standardized differences became smaller, often substantially so and always to <20%. The one exception was current smoking, whose weighted standardized difference increased from its crude value instead.

**Table 3:** Baseline characteristics of Groups 3A and 3B with crude and weighted standardized differences

Variable*	Group 3A (n = 112)	Group 3B (n = 242)	*P* value	Standardized difference	
				Crude	Weighted
Sociodemographic characteristics					
Age, years	58 (50, 67.5)	60 (52, 70)	.079	0.208	0.120
Males, n (%)	76 (67.86)	144 (59.50)	.132	0.172	0.002
Race‐ethnicity, n (%)			.512	0.035	0.046
Non‐hispanic white	20 (17.86)	40 (16.53)			
Hispanic	51 (45.54)	102 (42.15)			
Non‐hispanic black	25 (22.32)	49 (20.25)			
Other	16 (14.29)	51 (21.07)			
Summary socioeconomic score	−3.33 (−6.11, −1.48)	−2.38 (−5.44, −0.87)	.014	0.289	0.005
Risk factors					
Hypertension, n (%)	82 (73.21)	181 (74.79)	.752	0.036	0.014
Diabetes, n (%)	92 (82.14)	147 (60.74)	<.001	0.466	0.056
Dyslipidaemia, n (%)	69 (61.61)	151 (62.40)	.887	0.016	0.015
Cocaine use, n (%)	8 (7.14)	7 (2.89)	.087	0.211	0.006
Current smoker, n (%)	37 (33.04)	70 (28.93)	.434	0.089	0.180
Heavy alcohol use, n (%)	11 (9.8)	15 (6.2)	.224	0.139	0.043
Family history of CAD, n (%)	31 (28.97)	76 (31.40)	.649	0.053	0.113
Prior CVD, n (%)	24 (21.43)	64 (26.45)	.309	0.116	0.005
Prior HF, n (%)	7 (43.75)	9 (3.72)	.286	0.122	0.001
HIV infected, n (%)	3 (2.68)	2 (0.83)	.331	0.157	0.014
Home medications, n (%)					
Aspirin	43 (38.39)	85 (35.12)	.552	0.068	0.049
Beta‐blocker	33 (29.46)	77 (31.82)	.666	0.050	0.005
Calcium channel blocker	19 (16.96)	49 (20.25)	.466	0.083	0.007
RAAS antagonist	48 (42.86)	87 (35.95)	.213	0.142	0.103
Statin	43 (38.39)	83 (34.30)	.454	0.085	0.009
Thienopyridine	6 (5.36)	31 (12.81)	.033	0.244	0.054
Diabetes home medications, n (%)					
Oral hypoglycaemics OR insulin	75 (66.96)	126 (52.07)	.009	0.303	0.063
Admission findings					
Body mass index, kg/m^2^	28.5 (24.5, 32.2)	28.9 (25.9, 32.3)	.378	0.163	0.077
Systolic blood pressure, mm Hg	142 (118, 162)	139 (120, 158)	.695	0.043	0.028
Diastolic blood pressure, mm Hg	81 (67, 96)	80 (68, 94)	.549	0.099	0.060
Heart rate, beats per minute	83 (73, 96)	81 (70, 94)	.476	0.047	0.076
Killip class, n (%)			.446	0.131	0.077
I	97 (86.61)	219 (90.50)			
II	9 (8.04)	16 (6.61)			
III	6 (5.36)	7 (2.89)			
Left bundle branch block, n (%)	1 (0.89)	4 (1.65)	1.000	0.064	0.062
Non‐sinus rhythm, n (%)	3 (2.68)	18 (7.44)	.078	0.202	0.147
Presenting hospital, n (%)			<.001	0.613	0.044
Moses	78 (69.64)	97 (40.08)			
Weiler	34 (30.36)	145 (59.92)			
Transfer from another facility, n (%)	34 (30.36)	50 (20.66)	.046	0.229	0.057
Initial glucose, mg/dL	298 (242, 344)	234 (199, 291)	<.001	0.529	0.050
Initial WBC, 1000 per μL	11.3 (9.0, 14.5)	10.8 (8.6, 13.5)	.164	0.151	0.020
Initial serum creatinine, mg/dL	1.0 (0.8, 1.2)	0.9 (0.8, 1.2)	.635	0.067	0.046

Abbreviations: CAD, coronary artery disease; CIIT, continuous insulin infusion therapy; CVD, cardiovascular disease; HF, heart failure; HIV, human immunodeficiency virus; RAAS, renin‐angiotensin‐aldosterone system; WBC, white blood cell count; Group 3A = intial glucose ≥180 mg/dL and CIIT; Group 3B = intial glucose ≥180 mg/dL and no CIIT.

* Median and interquartile range for continuous variables.

**Figure 1:**
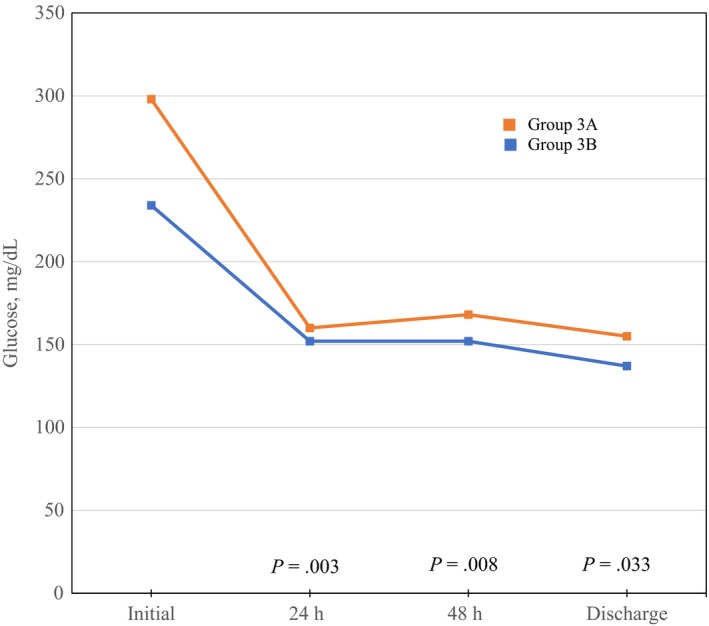
Median glucose values for the Groups 3A and 3B. The *P* value at each time point is for the difference between absolute drop from the initial glucose between the two groups. Group 3A = Intial glucose ≥180 mg/dL and CIIT; Group 3B = Intial glucose ≥180 mg/dL and no CIIT. CIIT = continuous insulin infusion therapy

Figure 1 shows the median levels for blood glucose for patients in Groups 3A and 3B, along with statistical comparisons of the change in these values over time in relation to those on admission. The absolute declines in glucose levels at 24, 48 hours and by the time of discharge, as compared with admission levels, were significantly greater in the Group 3A vs Group 3B.

**Figure 2:**
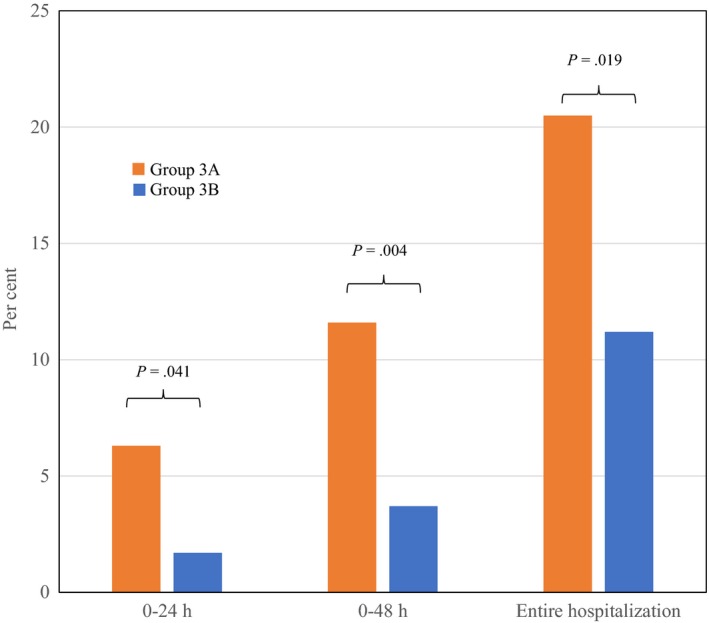
Proportion of patients suffering from hypoglycaemia at different time points between Groups 3A and 3B. Group 3A = intial glucose ≥180 mg/dL and CIIT; Group 3B = intial glucose ≥180 mg/dL and no CIIT. CIIT = continuous insulin infusion therapy

Figure 2 shows the proportions of Group 3 patients who experienced hypoglycaemic episodes (blood glucose ≤70 mg/dL) at various time points during the index hospitalization. The proportion of patients with a hypoglycaemic episode was significantly greater at all time points in Group 3A than Group 3B. Among patients who suffered hypoglycaemic episodes during their index hospitalization, there were 2 (8.7%) in‐hospital deaths in Group 3A and none in Group 3B (*P* = .207). In Group 3 patients, the occurrence of hypoglycaemia was not related to death (HR 1.05, 95% CI 0.53, 2.09), death or any readmission (HR 1.10, 95% CI 0.76, 1.60), or death or CVD readmission (HR 0.91, 95% CI 0.51, 1.30) during the full period of follow‐up in models adjusting for demographic factors and CIIT status.

### 3.4 Mortality and rehospitalization by CIIT status

Table 4 shows the crude and weighted numbers of events and crude and weighted risk estimates for death, death or any readmission and death or CVD readmission at different time points for Groups 3A and 3B. In crude analyses, there were significant associations with in‐hospital and 1‐year mortality, for which patients receiving CIIT had >3‐fold and >2‐fold increased risks, respectively, compared to those not receiving CIIT. These findings were similar after inverse‐probability‐of‐treatment weighting using the PS, although the risk of in‐hospital mortality became marginally nonsignificant. There were again no significant differences between the CIIT and no‐CIIT groups with respect to remaining time points and outcomes. Looking at STEMIs until December 2009, when lower glucose targets were advocated, there were no significant differences in the number of events [n = 11 (2.5%) vs n = 2 (3.9%) in‐hospital deaths, *P* = 1.0; and n = 3 (7.5%) vs n = 4 (7.7%) 1‐year deaths, *P* = 1.0] in the CIIT vs no CIIT groups.

**Table 4:** Outcomes for Groups 3A and 3B

Event	Events in Group 3A, n (%)	Events in GROUP 3B, n (%)	Crude risk estimates		Weighted events in Group 3A, n (%)	Weighted events in Group 3B, n (%)	Weighted Risk Estimates	
			Risk Estimate* (95% CI)^#^	*P* value			Risk Estimate* (95% CI)^#^	*P* value
Death								
In‐hospital	9 (8.0)	6 (2.5)	3.27 (1.18, 9.03)	.022	22 (6.3)	7 (2.2)	3.23 (0.94, 11.06)	.063
At 30 days	8 (7.1)	10 (4.1)	1.72 (0.69, 4.28)	.243	16 (4.5)	12 (3.6)	1.30 (0.44, 3.81)	.631
At 1 year	15 (13.4)	15 (6.2)	2.18 (1.09, 4.32)	.026	41 (11.8)	19 (5.7)	2.26 (1.02, 4.98)	.044
Entire follow‐up	26 (23.2)	35 (14.5)	1.42 (0.84, 2.39)	.191	87 (24.9)	44 (13.2)	1.56 (0.84, 2.91)	.162
Death or any readmission								
30 days	19 (17.0)	36 (14.9)	1.13 (0.68, 1.88)	.645	42 (12.1)	48 (14.2)	0.84 (0.45, 1.58)	.596
At 1 year	51 (45.5)	98 (40.5)	1.13 (0.87, 1.45)	.354	141 (40.3)	138 (41.0)	1.00 (0.69, 1.43)	.995
Entire follow‐up	77 (68.8)	140 (57.9)	1.19 (0.91, 1.58)	.205	215 (61.4)	193 (57.3)	0.93 (0.68, 1.43)	.929
Death or CVD readmission								
At 30 days	15 (13.4)	24 (9.9)	1.34 (0.73, 2.46)	.341	32 (9.1)	32 (9.6)	0.97 (0.47, 2.00)	.941
At 1 year	37 (33.0)	66 (27.3)	1.22 (0.87, 1.70)	.246	114 (32.6)	86 (25.6)	1.30 (0.84, 2.01)	.237
Entire follow‐up	59 (52.7)	97 (40.1)	1.31 (0.94, 1.80)	.107	168 (48.1)	126 (37.5)	1.27 (0.82, 1.97)	.288

Abbreviations: CI, confidence interval; CIIT, continuous insulin infusion therapy; CVD, cardiovascular disease; Group 3A = intial glucose ≥180 mg/dL and CIIT; Group 3B = intial glucose ≥180 mg/dL and no CIIT.

* All risk estimates are risk ratios except for those corresponding to comparisons through follow‐up, which are hazard ratios.

^#^ Group 3B is the referent and all models adjust for smoking status.

### 3.5 Stress hyperglycaemia

Fifty‐two patients met criteria for stress hyperglycaemia. As compared with 411 patients without known diabetes and initial glucose level <180 mg/dL, patients with stress hyperglycaemia were older, less often male, more frequently showed dyslipidaemia, had higher mean levels of cardiac biomarkers and exhibited a higher TIMI risk score (data not shown). There were no statistically significant differences in crude proportions or incidence rates for death, death or any readmission, or death or CVD readmission between the stress hyperglycaemia and the normal glycemic groups, or between stress hyperglycaemic and hyperglycaemic diabetic patients (data not shown).

## 4 DISCUSSION

### 4.1 Main findings

This investigation of acute STEMI patients in a socioeconomically disadvantaged urban area focusing specifically on hyperglycaemia and the impact of CIIT on associated short‐ and long‐term outcomes yielded several notable findings. First, admission hyperglycaemia was very common, affecting three‐fifths of the entire cohort. In more than half of such patients, hyperglycaemia was pronounced. Such pronounced hyperglycaemia was associated with female sex, black and Hispanic race‐ethnicity, and lower socioeconomic score, as well as higher TIMI risk score, LAD as the culprit lesion and lower LVEF. Second, patients with pronounced hyperglycaemia had correspondingly higher adjusted risks of death and combined death or CVD readmission at 1 year and through the duration of follow‐up, as compared to patients with normal glucose regulation. Third, fewer than one‐third of patients with pronounced hyperglycaemia received CIIT, a treatment that was applied to those with generally more severe, or more commonly pre‐existing diabetes than those who did not. Patients who received CIIT (Group 3A) had >2‐ to 3‐fold increased risks for in‐hospital and 1‐year mortality than those who did not receive CIIT (Group 3B) in crude analyses, increased risks that were virtually unchanged after IPTW. No other differences were observed in either risk of death at other time points or death and readmission at any time point. Those treated with CIIT also had more than twice the frequency of hypoglycaemia as compared with their non‐CIIT counterparts.

### 4.2 Acute STEMI and hyperglycaemia: determinants and outcomes

The present study contributes new information concerning the burden of STEMI‐related glycemic dysregulation in disadvantaged populations in contemporary practice, particularly among Hispanics. MHS serves Bronx County, the poorest in New York State, making the current STEMI registry quite distinct from the population included in a large nationwide sample. Our cohort included a large proportion of patients of Hispanic ethnicity (40% vs 5% in the National Cardiovascular Data Registry 2001 Report).^30^ Almost 60% of the sample was Hispanic or African American, and the median summary socioeconomic score overall was well within the bottom third reported in a population‐based study.^23^ The frequency of diabetes by history in our sample was 25% higher than previously documented for STEMI patients nationwide (32.6% vs 24.4%).^30^ This is consistent with other multi‐centre registries, which have documented higher prevalence of diabetes among blacks and Hispanics presenting with AMI.^31^, ^32^ Moreover, the Bronx Hispanic population is mostly of Puerto Rican and Dominican descent, setting it apart from previous studies reflecting Hispanics of Mexican background.^33^

Our study, however, provides details regarding the extent of STEMI‐related hyperglycaemia, showing it to be over 20% more common than previously reported in randomized trials (60% vs 47%).^34^ Such hyperglycaemia was pronounced in approximately one‐third of the sample, more commonly occurring in women, race‐ethnic minority groups and those with lower socioeconomic score. Patients with pronounced hyperglycaemia tended to have worse MI features, including an LAD culprit and worse LVEF. We did not, however, detect higher risk of adverse clinical outcomes in our patients with stress hyperglycaemia, as reported elsewhere, perhaps having to do with our exclusion of patients with cardiogenic shock.^35^ Still, patients with pronounced hyperglycaemia exhibited a significantly increased risk of adverse clinical events at follow‐up, as documented in other studies.^1^, ^2^, ^3^ This is likely attributable to the more severe and long‐standing glucose dysregulation in most of these patients and associated comorbidities observed in this group, coupled with disadvantages associated with their lower socioeconomic status regarding treatment adherence and access to care.

Notably, just over one‐third of patients with pronounced hyperglycaemia, most of whom had diabetes, were on aspirin, RAAS antagonists, or statins on admission, medications that would be indicated in a larger proportion of this sample. At discharge, over 25% of participants were not on RAAS antagonists or antihyperglycaemic therapy. These shortfalls in therapy attest to missed opportunities for primary or secondary prevention in this high‐risk population. Such data are currently being directed towards quality improvement efforts at MHS, but also underscore the need for greater outreach programmes for primary prevention in such underserved populations to improve uptake of guideline‐recommended medical therapies.^18^, ^36^

### 4.3 Impact of CIIT

Despite an MHS initiative encouraging institution of a CIIT protocol in acute STEMI patients, adoption of the protocol was limited to only a minority of registry patients.^17^, ^18^, ^37^ Selection of the protocol was reserved to patients with more profound glycemic abnormalities used preferentially at one of the sites, and more frequently among patients with lower socioeconomic score or those transferred from an outside facility. Use of CIIT tended to achieve faster glucose lowering as compared with usual care and was more frequently associated with hypoglycaemic episodes, yet blood glucose levels remained higher than target in many patients up to and including the time of discharge.

Notably, CIIT use was associated with marked risk increases for in‐hospital and 1‐year mortality, with risk estimates persisting even after IPTW eliminated substantive differences in measured risk factors. These differences were not seen with respect to 30‐day or longer‐term mortality, nor were any differences noted for death and readmission for any cause or cardiovascular causes. Our findings need to be interpreted in light of the modest size of our sample and attendant events, which led to wide confidence intervals and limited power to detect clinically meaningful associations. Although we successfully reduced the magnitude of intergroup differences between those who did and did not receive CIIT, residual confounding for unmeasured factors that would act to heighten mortality in the CIIT group could still account for the observed associations. But the signal for increased risk and its persistence in the weighted analysis does raise questions about the benefit‐to‐risk ratio of CIIT, especially in view of the higher incidence of hypoglycaemia observed for those receiving this treatment.

We were not able to detect an association between CIIT‐related hypoglycaemic episodes and untoward outcomes, or to meaningfully assess the impact of the lower initiation and treatment thresholds for glucose applied in the first 2‐years of the study period. Other studies have documented the adverse consequences of hypoglycaemia in clinical care, however, and guidelines emphasize the imperative to avoid hypoglycaemia in the management of STEMI‐related hyperglycaemia for this reason.^18^, ^38^, ^39^ Hence, although the basis for the increased CIIT‐associated mortality risk documented here is uncertain, the present findings strike an added note of caution about CIIT use and highlight the need for large‐scale and preferably randomized approaches to defining its potential impact.

### 4.4 Strengths and limitations

Our study has a number of strengths. It focuses on a disadvantaged population that remains understudied as relates to metabolic dysregulation in the setting of acute STEMI. It leverages inclusive data from MHS, the principal care provider for Bronx County, New York, and employs its clinical and administrative information systems to capture multi‐layer data pertaining to STEMI care and outcomes. Also, our study includes details on social habits, such as alcohol and cocaine use, and HIV status, that are important in this context but often not available in larger registries.

Among its limitations, the study sample is of moderate size. Classification of glycemic categories was based on the initial glucose, irrespective of fasting status, a standard approach in the AMI setting that would tend to bias the comparisons of interest towards the null hypothesis. Although the NDI afforded comprehensive assessment of mortality, the study was only able to capture rehospitalizations to MHS and NBHN. This may have led to underascertainment of hospitalizations and potentially misclassification bias of uncertain direction. However, results for rehospitalization were broadly similar to patterns seen for mortality, suggesting that such bias, if any, did not majorly influence our findings.

## 5 CONCLUSIONS

In this disadvantaged urban population, glycemic abnormalities were highly prevalent in the context of acute STEMI, more common among race‐ethnic minorities and those with lower socioeconomic status, and associated with increased risk of poor outcomes, as compared with those reported in other cohorts. Despite its potential advantages, uptake of a recommended CIIT protocol was limited, reserved largely for patients with previously treated diabetes and marked glycemic abnormalities, and was associated with significantly or near‐significantly increased risks of in‐hospital and 1‐year mortality even after applying PS‐weighting to account for treatment group differences. The extent to which such increased risks related to potential adverse effects of CIIT or residual confounding cannot be determined by our quasi‐experimental design in this moderate‐sized sample. Hence, our findings underscore the need for larger evaluations, and particularly randomized trials, to address this question. In view of the modern epidemics of obesity and diabetes, and their disproportionate impact on socioeconomically disadvantaged race‐ethnic minorities, this remains an issue of high priority for improving care and remedying health disparities in AMI outcomes.

## CONFLICT OF INTEREST

JRK reports stock ownership in Bristol‐Myers Squibb, Johnson & Johnson, Medtronic, Merck, and Pfizer. SGS reports stock ownership in Amgen Inc, Johnson & Johnson and GlaxoSmithKline.

## AUTHORS' CONTRIBUTIONS

VS and JS developed the study protocol and, along with JRK, formulated the study design. JLB, TS, MJP, SH, AC and NP contributed in data collection. SGS and XX conducted analysis of the data. SGS, XX, JS and JRK performed data interpretation. SGS, JS and JRK drafted the manuscript. All authors participated in review and revision of the manuscript for important intellectual content.

## ETHICS APPROVAL AND CONSENT TO PARTICIPATE

The STEMI registry protocol was approved by the Institutional Review Board of the Albert Einstein College of Medicine. Informed Consent was obtained from all participants.

## CONSENT FOR PUBLICATION

Not applicable.

## Data Availability

The data sets used and/or analysed during the current study are available from the corresponding author on reasonable request.
